# The Estradiol Synthesis Inhibitor Formestane Diminishes the Ability of Sevoflurane to Induce Neurodevelopmental Abnormalities in Male Rats

**DOI:** 10.3389/fnsys.2020.546531

**Published:** 2020-09-04

**Authors:** Jie Wang, Baofeng Yang, Lingsha Ju, Jiaojiao Yang, Andrea Allen, Jiaqiang Zhang, Anatoly E. Martynyuk

**Affiliations:** ^1^Department of Anesthesiology and Perioperative Medicine, Henan Provincial People’s Hospital, People’s Hospital of Zhengzhou University, People’s Hospital of Henan University, Zhengzhou, China; ^2^Department of Anesthesiology, University of Florida College of Medicine, Gainesville, FL, United States; ^3^Department of Anesthesiology and Perioperative Medicine, Affiliated, Cancer Hospital of Zhengzhou University, Zhengzhou, China; ^4^The McKnight Brain Institute, University of Florida College of Medicine, Gainesville, FL, United States

**Keywords:** sevoflurane, EEG seizures, behavioral abnormalities, corticosterone, estradiol, excitatory GABA_*A*_ receptor signaling

## Abstract

**Background:**

In rodents, the period of increased vulnerability to the developmental effects of general anesthetics coincides with the period of age-specific organizing (masculinizing) effects of the major female sex hormone 17β-estradiol (E2) in the male brain and excitatory GABA type A receptor (GABA_*A*_R) signaling. We studied whether E2 synthesis and excitatory GABA_*A*_R signaling are involved in the mediation of the developmental effects of sevoflurane in male rats.

**Methods:**

Male Sprague-Dawley rats were pretreated with the inhibitors of E2 synthesis, formestane, or the Na^+^-K^+^-2Cl^–^ (NKCC1) Cl^–^ importer, bumetanide, prior to sevoflurane exposure for 6 h on postnatal (P) day 4, P5, or P6. We tested whether a subsequent exposure of these rats to sevoflurane on P∼10 would cause electroencephalography (EEG)-detectable seizures. We also evaluated their behavior during the elevated plus maze (EPM) test on P∼60, prepulse inhibition (PPI) of acoustic startle responses on P∼70, and corticosterone secretion to physical restraint on P∼80.

**Results:**

The rats neonatally exposed to sevoflurane responded to repeated exposure to sevoflurane with increased EEG-detectable seizures (*F*_(__3_,_24__)_ = 7.445, *P* = 0.001) and exhibited deficiencies during the EPM (*F*_(__3_,_55__)_ = 4.397, *P* = 0.008) and PPI (*F*_(__3_,_110__)_ = 5.222, *P* = 0.003) tests. They also responded to physical restraint with heightened secretion of corticosterone (*F*_(__3_,_16__)_ = 11.906, *P* < 0.001). These parameters in the sevoflurane-exposed rats that were pretreated with formestane or bumetanide were not different from those in the control rats.

**Conclusion:**

These results, along with previously published data, suggest that sevoflurane-enhanced E2 synthesis and excitatory GABA_*A*_R signaling at the time of sevoflurane anesthesia are involved in the mediation of the neurodevelopmental effects of the anesthetic in male rats.

## Introduction

According to some surveys, 1.5 million children may be exposed to anesthesia during their first 12 months of life each year in the United States (DeFrances et al., 2007; Servick, 2014). Human and animal studies support the notion that prolonged and/or repeated exposure to general anesthesia early in life may initiate neurodevelopmental abnormalities (Vutskits and Xie, 2016; Lin et al., 2017). Such concern led to the United States Food and Drug Administration safety warning on the use of general anesthetics in pregnant women and young children (Us Food and Drug Administration, 2017). In an attempt to improve the safety of pediatric anesthesia, numerous studies have proposed various mediating mechanisms of the adverse effects of general anesthetics in neonatal animal models, further confirming the mechanistic complexity of this phenomenon (Vutskits and Xie, 2016; Lin et al., 2017). Our approach in this field is based on an assumption that the developing brain is especially vulnerable to the deleterious effects of general anesthetics because, at least in part, the anesthetics exert their adverse effects by affecting mechanisms specific to this age period (Edwards et al., 2010; Tan et al., 2014; Xu et al., 2015; Zhang et al., 2016; Ju et al., 2017, 2018, 2019).

Among such age-dependent mechanisms is depolarizing/excitatory GABA type A receptor (GABA_*A*_R) signaling (Farrant and Kaila, 2007; Nuñez and McCarthy, 2009; Ben-Ari et al., 2012; Dehorter et al., 2012; Ben-Ari, 2014; Ito, 2016), a major substrate for the otherwise inhibitory effects of GABAergic anesthetics at more mature age (Kotani and Akaike, 2013; Antkowiak and Rudolph, 2016). The GABA_*A*_R signaling is mainly excitatory in the rostral regions of the brain during early life due to elevated levels of intraneuronal Cl^–^ maintained by relatively low and high levels of the K^+^-2Cl^–^ (KCC2) Cl^–^ exporter and the Na^+^-K^+^-2Cl^–^ (NKCC1) Cl^–^ importer, respectively (Farrant and Kaila, 2007; Nuñez and McCarthy, 2009; Ben-Ari et al., 2012; Dehorter et al., 2012; Ben-Ari, 2014; Ito, 2016). During the second postnatal week in rodents and during the first year of life in humans, GABA_*A*_R signaling gradually becomes inhibitory, primarily due to age-dependent increases in KCC2 (Dzhala et al., 2005; Farrant and Kaila, 2007; Nuñez and McCarthy, 2009; Ben-Ari et al., 2012; Dehorter et al., 2012; Ben-Ari, 2014; Ito, 2016). The magnitude of excitatory GABA_*A*_R signaling and the proper timing of its transition from excitatory to inhibitory are essential for normal brain development and functioning. The loop diuretic bumetanide, when it is used at low doses, is currently the most selective inhibitor of NKCC1 (Dzhala et al., 2005). Our lab and others have demonstrated that pretreatment of neonatal rats with bumetanide prior to anesthesia exposure ameliorated many anesthetic-induced acute and long-term abnormalities (Edwards et al., 2010; Tan et al., 2014; Xu et al., 2015; Liu et al., 2016; Zhang et al., 2016; Ju et al., 2017). The alleviating effects of NKCC1 inhibition prior to anesthetic exposure suggest that anesthetic-exacerbated excitatory GABA_*A*_R signaling may be involved in the mediation of the developmental effects of GABAergic anesthetics.

Also, the sex steroid hormone 17β-estradiol (E2) exerts organizational (persistent) effects in the male brain during a “sensitive period” that in rodents occurs almost during the same age period when they are most vulnerable to the developmental effects of general anesthetics (McCarthy and Arnold, 2011; Stratmann, 2011). The sensitive period starts prenatally with the onset of testis-produced testosterone, which is then converted in the male brain to E2 by the enzyme aromatase. The sensitive period ends during the second postnatal week when females become insensitive to the masculinizing effects of exogenous sex hormones. E2 can also be synthesized in the brain *de novo* in both sexes (McCarthy and Arnold, 2011). We have previously demonstrated that E2 may exacerbate the acute adverse effects of sevoflurane in neonatal rats through a direct potentiation of excitatory GABA_*A*_R signaling at the time of anesthesia (Zhang et al., 2016). Because E2 can exert persistent organizing effects in the male brain during this age period, here we investigated whether E2 can also be involved in the mediation of the long-term neurodevelopmental effects of sevoflurane in male rats. We have done so by assessing the effects of the E2 synthesis inhibitor formestane, administered prior to sevoflurane exposure, on the anesthetic’s abilities to expand the window of vulnerability to repeated exposure to sevoflurane. We also assessed how formestane affected sevoflurane’s ability to induce behavioral abnormalities and to impair functioning of the hypothalamic-pituitary-adrenal (HPA) axis. Rats, pretreated with the NKCC1 inhibitor bumetanide prior to neonatal exposure to sevoflurane, were used as a positive control.

## Materials and Methods

### Animals

All experimental procedures were approved by the University of Florida Institutional Animal Care and Use Committee (Gainesville, FL, United States). All animal experiments complied with the ARRIVE guidelines and were carried out in accordance with the National Institutes of Health guide for the care and use of Laboratory animals (NIH Publications No. 8023, revised 1978). Sprague-Dawley rats were studied. Animals were housed under controlled illumination (12-h light/dark cycle, lights on at 7 am) and temperature (23–24°C) with free access to food and water. Within 24 h of delivery, litters were culled to 12 pups. At 21 days old, pups were weaned and housed in sex-matched groups of two for the remainder of the study. To control for litter variability, several pups from different litters were used for each treatment condition. Multiple sets of animals were used in the experiments. The data reported in this study were collected from 87 male rats.

### Treatment Groups

The postnatal day 4 (P4), P5, or P6 male rat pups were kept in a temperature-controlled chamber to maintain body temperature at +37°C with a continuous supply of 30% oxygen in air (1.5 L/min) during anesthesia: 6% sevoflurane for 3 min for anesthesia induction and then 2.1% sevoflurane for 357 min for anesthesia maintenance. All animals except those in the Control group received a subcutaneous injection of saline (1 mL/100 g) at 2 and 4 h of anesthesia with sevoflurane to prevent dehydration. The E2 synthesis inhibitor formestane (2 mg/kg, subcutaneous injection) or the Na^+^-K^+^-2Cl^–^ cotransporter inhibitor bumetanide (1.82 mg/kg, intraperitoneal injection) was administered to animals 30 min prior to anesthesia with sevoflurane (the formestane + sevoflurane group and the bumetanide + sevoflurane group, respectively). The animals in the vehicle + sevoflurane group were injected with equal volumes of vehicle for formestane or bumetanide (the vehicle + sevoflurane group). The control animals (the control group) were not subjected to anesthesia and remained with their dams. The primary argument against the use of a control group for maternal separation during sevoflurane anesthesia was that anesthetized pups do not experience separation stress, while 6 h of maternal separation is a stressor that could serve as a confounding factor (Xu et al., 2015). Gas monitoring was performed using a calibrated Datex side stream analyzer (Datex-Ohmeda, Helsinki, Finland), which was sampled from the interior of the animal chamber. Sevoflurane at 2.1% is near the 0.6 minimum alveolar concentration for P4 to P6 rats (Orliaguet et al., 2001). At the dose of 2.1% sevoflurane, the pups did not exhibit a righting reflex. Previously, we have shown that blood glucose and gas levels after 2.1% sevoflurane anesthesia for 6 h were in the normal range, while higher doses of sevoflurane (e.g., 2.9%) may cause respiratory depression in spontaneously breathing rats (Edwards et al., 2010).

**FIGURE 1:**
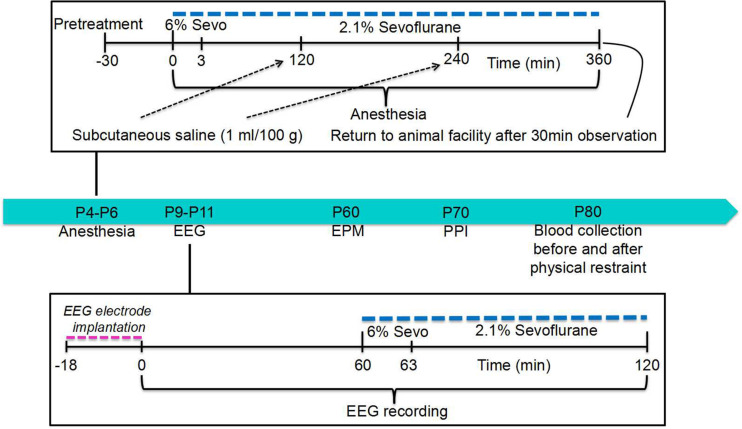
Illustration of the study design. See text for details.

A subset of the rat pups from all experimental groups was used to test whether anesthesia with sevoflurane for 6 h on P4, P5, or P6 widens the age window during which repeated exposure to sevoflurane can cause electroencephalography (EEG)-detectable seizures. This subset was also used to test how sevoflurane’s effect is altered by pretreatments with formestane or bumetanide prior to sevoflurane exposure on P4, P5, or P6. For this purpose, the P9, P10, or P11 rats from all four experimental groups, including the control group, underwent EEG recordings for 1 h of baseline activity and for another hour during the sevoflurane exposure (see section “EEG Recordings” and Figure 1 for illustration of experimental design). We have previously shown that the ability of sevoflurane to cause EEG-detectable seizures in naïve rats (not neonatally exposed to sevoflurane) during this age period is drastically decreased (Edwards et al., 2010). The rest of the rats were used to evaluate the effects of pretreatments with formestane or bumetanide prior to sevoflurane exposure on P4, P5, or P6 on the ability of the anesthetic to induce behavioral deficiencies and abnormal functioning of the HPA axis in adulthood. These rats were sequentially evaluated in the elevated plus maze (EPM) on P∼60 and prepulse inhibition (PPI) of the acoustic startle response on P∼70, as well as for their corticosterone responses to stress caused by physical restraint for 30 min on P∼80.

### EEG Recordings

To measure EEG activity, the P9, P10, or P11 rat pups were instrumented for EEG recording as described previously (Edwards et al., 2010; Zhang et al., 2016). In brief, during a 12- to 18-min minor surgical procedure performed under isoflurane anesthesia (1.6–2.0%), four electrodes were implanted in the bilateral occipital and frontal regions of the rat pup skull with the left frontal electrode serving as the reference electrode. EEG recordings were performed in a thermostat-controlled chamber to maintain body temperature at +37°C with a continuous supply of 30% oxygen in air (1.5 L/min) using an EEG/electromyogram system (Pinnacle Technology, Lawrence, KS, United States). Acquisition of the EEG was performed using the Sirenia software (Pinnacle Technology). The sampling interval per signal was 200 μs (5 kHz). The recordings started after the rats recovered from anesthesia for electrode implantation and lasted for 1 h before the initiation of anesthesia with sevoflurane and continued for another hour during anesthesia with 6% sevoflurane for 3 min for anesthesia induction and 2.1% sevoflurane for 57 min for anesthesia maintenance. Sirenia Score software (Pinnacle Technology) was used for the EEG data analysis. EEG patterns characterized by an amplitude of at least three times higher than baseline and rhythmic activity (>2 Hz) that lasted for at least 3 s and abruptly reverted to the baseline were defined as seizure-like EEG patterns. In most cases, these patterns started as high frequency–low amplitude activity that developed to increased amplitude and decreased frequency and then abruptly reverted to baseline activity. All parameters for EEG seizures such as the total duration, number of episodes, and average episode duration were calculated for the entire 60-min period of sevoflurane exposure. The investigators analyzing the EEGs were blinded to the experimental conditions, and all EEGs were reviewed by three independent reviewers.

### Assessment of Behavior in the EPM

The EPM studies were performed using an EPM apparatus and BIO-EPM 3C video tracking software (EB Instruments, Pinellas Park, FL, United States) during the light phase of the dark-light cycle as previously described by our laboratory (Xu et al., 2015). The maze consists of two opposing open (50 × 10 × 0.5 cm) and two enclosed (50 × 10 × 45 cm) arms elevated 75 cm above the floor, with a 0.5-cm edge on the open arms. Testing occurred during the light phase of the dark-light cycle. Animals were placed in the center square facing an open arm and were allowed to explore the maze for 5 min, at which time they were removed from the apparatus. During EPM testing, each rat’s behavior was recorded using BIO-EPM 3C video tracking software. The percentage of time spent in the open arms and the total distance traveled during 5 min of recording as an index of the locomotor activity were compared. If a fall occurred, the animal was removed from the study (one male rat from the vehicle + sevoflurane group was removed from the study for this reason).

### Measurements of the PPI of Startle

The PPI of startle tests were performed using an SR-Lab startle apparatus (San Diego Instruments, San Diego, CA, United States) as previously described by our laboratory (Cao et al., 2012; Xu et al., 2015). Testing occurred during the light phase of the dark-light cycle. At the beginning of every testing session, each animal was placed in the cylindrical animal enclosure and exposed to a 75-dB white noise background for a 5-min acclimation period. The acclimation period was followed by a test session consisting of five different trials: a 120 dB 40 ms pulse only; a 120 dB 40 ms pulse preceded by a 20-ms prepulse at 3, 6, and 12 dB above background; and a no-stimulus trial of background noise. The delay between the onset of the prepulse and the onset of the pulse was 100 ms. The trials were presented in pseudorandom order with variable inter-trial intervals averaging 15 s. The first four trials and the last three trials consisted of 120 dB pulse-only trials. All five types of trials were presented eight times, each in pseudorandom order after the first four and before the last three pulse-only trials. The %PPI for each prepulse level was calculated using the formula: %PPI = 100 × [(pulse alone) – (prepulse + pulse)]/pulse alone. Data were collected as *V*_*max*_ amplitude.

### Basal and Stress-Induced Levels of Corticosterone

To measure changes in serum levels of corticosterone in response to physical restraint, blood samples (∼300 μL) were collected using the “tail clip” method (Vahl et al., 2005) before (at rest) and at 10, 60, and 120 min after the restraint for 30 min as previously described by our laboratory (Ju et al., 2017). Physical restraint was administered using rodent holders (Kent Scientific Corporation, Torrington, CT, United States). Serum corticosterone was measured using commercial ELISA kits (Cayman Chemical Company, Ann Arbor, MI, United States) according to the manufacturer’s instructions.

### Drugs

Sevoflurane was manufactured by Fushimi-machi (Osaka, Japan). Bumetanide (Ben Venue Laboratories Inc., Bedford, OH, United States) was purchased from Bedford Laboratories^TM^ (Bedford, OH, United States) and formestane was acquired from Sigma-Aldrich (St. Louis, MO, United States).

### Statistical Analysis

Statistical analyses were conducted on raw data using SigmaPlot 12.5 software (Systat Software Inc., San Jose, CA, United States), which automatically checks if the data set meets test criteria (Shapiro-Wilk for normality test and Brown-Forsythe for equal variance test). Values are reported as mean ± SEM. To analyze data for EEG-detectable seizures and EPM, one-way analysis of variance (ANOVA) was used. A two-way repeated measures ANOVA was used to analyze the PPI data, with the treatment and prepulse intensity as independent variables. A two-way repeated measures ANOVA was used to analyze changes in the serum corticosterone levels at rest and at three time points after the restraint, with experimental groups and time as the independent variables. To assess the differences in total corticosterone concentrations, the area under the curve with respect to baseline (AUCg, levels of corticosterone at rest) was calculated and compared across experimental groups using one-way ANOVA. All multiple pairwise comparisons were done using the Fisher LSD method. *P* < 0.05 was considered significant. Statistical details are presented in the text and figure legends. The sample sizes in this study were based on previous experience with the same experimental techniques and measured parameters (Edwards et al., 2010; Tan et al., 2014; Xu et al., 2015; Zhang et al., 2016; Ju et al., 2017, 2018, 2019).

## Results

### Formestane or Bumetanide, Administered 30 min Prior to Anesthesia With Sevoflurane on P4, P5, or P6, Reduce the Ability of Sevoflurane to Cause EEG-Detectable Seizures on P9, P10, or P11 in Male Rats

**FIGURE 2:**
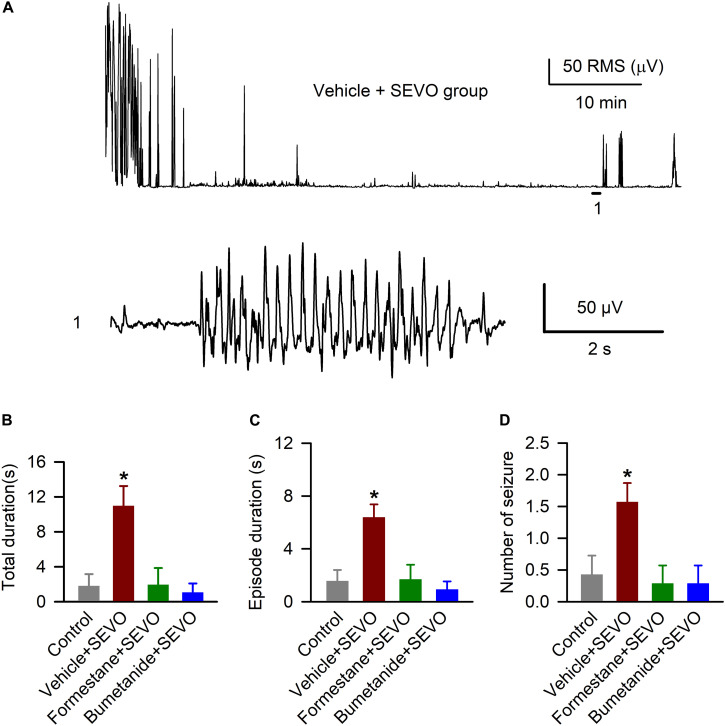
Formestane or bumetanide, administered 30 min prior to anesthesia with sevoflurane (SEVO) on postnatal day 4 (P4), P5, or P6, reduce the ability of sevoflurane to cause electroencephalography (EEG)-detectable seizures on P9, P10, or P11 in male rats. **(A)** Root mean square (RMS) of the EEG of a P10 male rat before and during sevoflurane exposure. The horizontal line marks the occurrence of a seizure episode (1); corresponding section of the electroencephalogram is shown at expanded time scale below. **(B–D)** Histograms showing total duration of seizure activity, average seizure episode duration, and number of seizure episodes during 1 h of anesthesia with sevoflurane in the P9, P10, or P11 male rats. Data are means ± SEM from 7 rats/group. *P < 0.05 vs all other groups.

We studied the ability of sevoflurane to cause EEG-detectable seizures in the P9, P10, or P11 male rats in all four experimental groups, i.e., the control rats and rats that were exposed to sevoflurane for 6 h on P4, P5, or P6 after pretreatment with the vehicle, formestane, or bumetanide. There was a significant between-subjects effect of treatment on P4, P5, or P6 on the P9, P10, or P11 exposure to sevoflurane-caused total duration of EEG-detectable seizures (*F*_(__3_,_24__)_ = 7.445, *P* ≤ 0.001; Figures 2A,B), duration of seizure episode (*F*_(__3_,_32__)_ = 8.487, *P* < 0.001; Figures 2A,C), and number of seizure episodes (*F*_(__3_,_24__)_ = 4.560, *P* = 0.012; Figures 2A,D). Specifically, the P9, P10, or P11 male rats in the vehicle + sevoflurane group had greater total duration (10.957 ± 2.278 s; *P* = 0.001), episode duration (6.392 ± 0.974 s; *P* < 0.001), and number of episodes of seizures (1.571 ± 0.297; *P* < 0.011) compared with all other treatment groups. The P9, P10, or P11 male rats in the formestane + sevoflurane group and the bumetanide + sevoflurane group were not different from male rats in the control group with respect to total duration [1.800 ± 1.367 s (the control group) and 1.929 ± 1.929 s (the formestane + sevoflurane group), *P* = 0.958; and 1.043 ± 1.043 s (the bumetanide + sevoflurane group), *P* = 0.759], episode duration [1.575 ± 0.830 s (the control group) and 1.688 ± 1.114 s (the formestane + sevoflurane group), *P* = 0.937; and 0.925 ± 0.163 s (the Bumetanide + sevoflurane group), *P* = 0.648] and number of episodes of EEG seizures [0.429 ± 0.297 (the control group) and 0.286 ± 0.286 (the formestane + sevoflurane group), *P* = 0.732; and 0.286 ± 0.286 (the bumetanide + sevoflurane group), *P* = 0.732].

### Formestane or Bumetanide, Administered to Male Rats 30 min Prior to Anesthesia With Sevoflurane on P4, P5, or P6, Reduce the Ability of Sevoflurane to Induce Abnormal Behavior During the EPM Test

**FIGURE 3:**
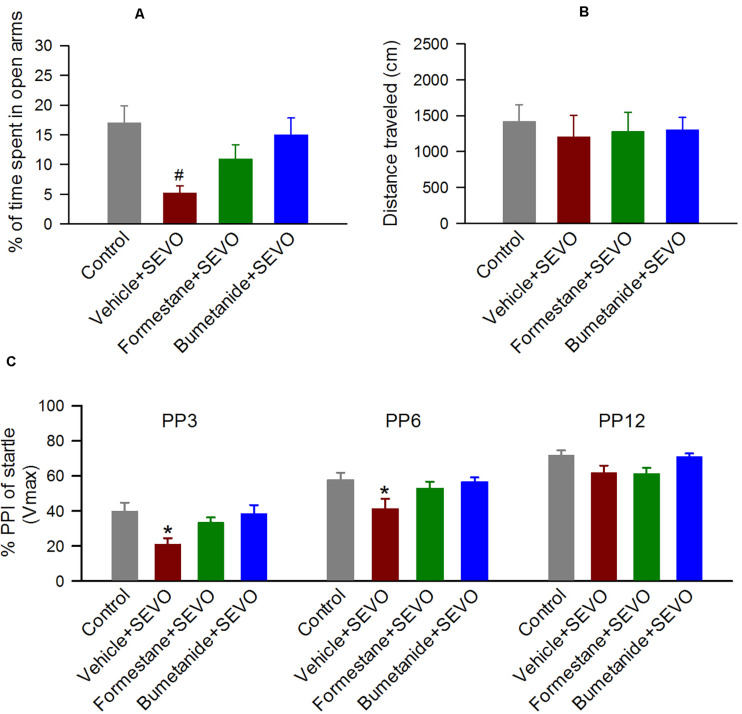
Formestane or bumetanide, administered 30 min prior to anesthesia with sevoflurane (SEVO) on postnatal day 4 (P4), P5, or P6, reduce the ability of sevoflurane to induce abnormal behavior during the elevated plus maze (EPM) and prepulse inhibition (PPI) of the acoustic startle response tests in male rats. **(A,B)** Histograms showing percent time spent in the open arms of the EPM and distance traveled by the P∼60 male rats during the test. Data are means ± SEM from 15 rats/group (14 male rats in the vehicle + sevoflurane group). ^#^*P* < 0.05 vs the control and bumetanide + sevoflurane groups. **(C)** Histogram showing %PPI of startle responses at prepulse intensity of 3 dB (PP3), 6 dB (PP6), and 12 dB (PP12) above background in the P∼70 male rats. Data are means ± SEM from 15 rats/group (14 male rats in the vehicle + sevoflurane group). *P < 0.05 vs. all other groups.

To investigate the effects of pretreatments with formestane or bumetanide prior to sevoflurane exposure for 6 h on P4, P5, or P6 on the ability of sevoflurane to induce increased anxiety-like behavior, the P∼60 rats were evaluated in the EPM behavioral paradigm. In the EPM test, there was a significant between-subjects effect of treatment on P4, P5, or P6 on the time the rats spent in the open arms (*F*_(__3_,_55__)_ = 4.397, *P* = 0.008; Figure 3A), but not on the distance that they traveled in the maze during the test (*F*_(__3_,_55__)_ = 1.941, *P* = 0.134; Figure 3B). Specifically, the P∼60 male rats in the vehicle + sevoflurane group spent a shorter time in the open arms (5.197 ± 1.214 s) compared with the Control group (17.016 ± 2.817 s, *P* = 0.001) and the bumetanide + sevoflurane group (15.009 ± 2.842 s, *P* = 0.007), but not with the formestane + sevoflurane group (10.947 ± 2.401 s, *P* = 0.107). However, the time that rats in the formestane + sevoflurane group and in the control group spent in the open arms was not different (*P* = 0.084).

### Formestane or Bumetanide, Administered to Male Rats 30 min Prior to Anesthesia With Sevoflurane on P4, P5, or P6, Reduce the Ability of Sevoflurane to Induce Impaired PPI of the Startle Responses

To investigate the effects of pretreatments with formestane or bumetanide prior to sevoflurane exposure for 6 h on P4, P5, or P6 on the ability of sevoflurane to induce impairment in sensorimotor gating function, the P∼70 male rats were evaluated for PPI of the acoustic startle responses. There was a significant between-subjects effect of treatment on P4, P5, or P6 on PPI of the startle responses (*F*_(__3_,_110__)_ = 5.222, *P* = 0.003; Figure 3C). Multiple pairwise comparisons found that male rats in the vehicle + sevoflurane group had reduced PPI of the startle response at prepulse intensities of 3 dB (39.874 ± 3.653 (the control group); 21.047 ± 3.781 (the vehicle + sevoflurane); 33.531 ± 3.653 (the formestane + sevoflurane group); and 38.529 ± 3.653 (the bumetanide + sevoflurane group); *P* ≤ 0.019 the vehicle + sevoflurane group vs all other groups) and 6 dB (57.752 ± 3.653 (the control group); 41.425 ± 3.781 (the vehicle + sevoflurane); 53.068 ± 3.653 (the formestane + sevoflurane group); 56.605 ± 3.653 (the bumetanide + sevoflurane group); *P* ≤ 0.029 the vehicle + sevoflurane group vs all other groups). The PPI of the startle responses in male rats in the formestane + sevoflurane group and the bumetanide + sevoflurane group were not different from those in male rats in the control group [*P* = 0.092 (the formestane + sevoflurane group) and *P* = 0.790 (the bumetanide + sevoflurane group)].

### Formestane or Bumetanide, Administered 30 min Prior to Anesthesia With Sevoflurane on P4, P5, or P6, Reduce the Ability of Sevoflurane to Induce Heightened Corticosterone Responses to Stress in Male Rats

**FIGURE 4:**
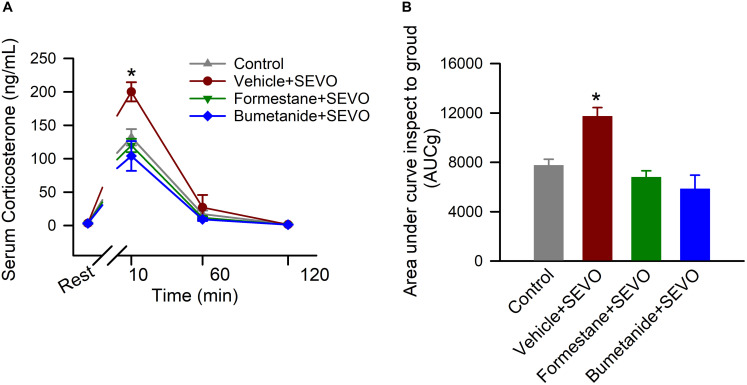
Formestane or bumetanide, administered 30 min prior to anesthesia with sevoflurane (SEVO) on postnatal day 4 (P4), P5, or P6, reduce the ability of sevoflurane to induce heightened corticosterone responses to stress in the P∼80 male rats. Plots showing the respective levels of serum corticosterone before physical restraint for 30 min and at 10, 60, and 120 min after the restraint **(A)** as well as the total corticosterone responses (measured as area under the curve). **(B)** Serum levels of corticosterone at rest were taken as baselines for calculations of the total corticosterone responses. Data are means ± SEM from 5 rats/treatment group. *P < 0.05 vs all other treatment groups.

To investigate the effects of pretreatments with formestane or bumetanide prior to sevoflurane exposure for 6 h on P4, P5, or P6 on the ability of sevoflurane to induce dysregulation of HPA axis functioning, the serum levels of corticosterone were measured in the P∼80 male rats at rest and at 10, 60, and 120 after physical restraint for 30 min. There was a significant between-subjects effect of treatment on P4, P5, or P6 on total serum levels of corticosterone after the physical restraint (*F*_(__3_,_16__)_ = 11.906, *P* < 0.001; Figures 4A,B). Multiple pairwise comparisons found that when compared with all other groups, rats in the vehicle + sevoflurane group had significantly higher levels of serum corticosterone [7753.233 ± 498.625 (the control group); 11731.961 ± 706.686 (the vehicle + sevoflurane group); 6792.044 ± 526.576 (the formestane + sevoflurane group); 5854.261 ± 1104.440 (the bumetanide + sevoflurane group); *P* ≤ 0.002 the vehicle + sevoflurane group vs all other groups]. These increases were due to higher levels of corticosterone at 10 min after the restraint [132.115 ± 9.172 ng/mL (the control group); 200.069 ± 9.172 ng/mL (the vehicle + sevoflurane group); 119.858 ± 9.172 ng/mL (the formestane + sevoflurane group); 104.091 ± 9.172 ng/mL (the bumetanide + sevoflurane group); *P* < 0. 001 the vehicle + sevoflurane group vs all other treatment groups]. The serum levels of corticosterone were similar in all groups before the restraint [2.987 ± 9.172 ng/mL (the control group); 3.197 ± 9.172 ng/mL (the vehicle + sevoflurane group); 3.057 ± 9.172 ng/mL (the formestane + sevoflurane group); 3.203 ± 9.172 ng/mL (the bumetanide + sevoflurane group); *P* = 0.991], 60 min post-restraint [17.200 ± 9.172 (the control group); 27.060 ± 9.172 ng/mL (the vehicle + sevoflurane group); 11.704 ± 9.172 ng/mL (the formestane + sevoflurane group); 9.164 ± 9.172 ng/mL (the bumetanide + sevoflurane group); *P* = 0.596] and 120 min post-restraint [1.857 ± 9.172 ng/mL (the control group); 1.365 ± 9.172 ng/mL (the vehicle + sevoflurane group); 1.186 ± 9.172 ng/mL (the formestane + sevoflurane group); 1.192 ± 9.172 ng/mL (the bumetanide + sevoflurane group); *P* = 0.094]. The post-restraint serum levels of corticosterone in the P∼80 male rats pretreated with formestane or bumetanide prior to sevoflurane exposure on P4, P5, or P6 were not different from those in the control group [*P* = 0.378 (the formestane + sevoflurane group) and *P* = 0.092 (the bumetanide + sevoflurane group)].

## Discussion

The novel finding of this study is that sevoflurane administered to neonatal male rats can broaden the age window during which male rats are vulnerable to adverse effects caused by subsequent administration of the anesthetic. We found that sevoflurane administered to P9, P10, or P11 male rats caused significantly greater EEG-detectable seizures in rats that were neonatally exposed to 6 h of anesthesia with sevoflurane than in their control counterparts that were not neonatally exposed to the anesthetic. If neonatal sevoflurane exposure similarly makes rats more vulnerable to other neurodevelopmental effects of sevoflurane during subsequent exposures, this finding may have important translational applicability. Understanding the effects of repeated anesthetic exposures is important because repeated operations are frequently required for complex conditions in those who had initial operations as preemies, neonates, or infants (Jevtovic-Todorovic, 2016). Additionally, repeated exposures are one of the prognostic factors for more severe outcomes in human studies (Wilder et al., 2009; Sprung et al., 2012). The alleviating effects of pretreatments with the E2 synthesis inhibitor formestane or the NKCC1 inhibitor bumetanide suggest that sevoflurane acts through the E2-dependent and depolarizing/excitatory GABA_*A*_R signaling-dependent mechanisms to induce such effects in male rats. Similar E2-dependent and depolarizing/excitatory GABA_*A*_R signaling-dependent mechanisms may be involved in the long-term behavioral deficiencies and exacerbated corticosterone responses to stress induced by neonatal exposure to sevoflurane. Importantly, sevoflurane may act through potentiation of synthesis of E2 to induce long-term neurodevelopmental abnormalities during the age period when E2 is known to exert lasting organizational effects in the male rodent brain (E2-regulated brain masculinization).

Similar preventive effects of pretreatments with formestane and bumetanide suggest that synthesis of E2 and depolarizing/excitatory GABA_*A*_R signaling enhanced by neonatal exposure to sevoflurane may act in concert to mediate the neurodevelopmental effects of the anesthetic. This possibility is supported by our recently published findings showing that exogenous E2, directly applied to brain tissue slices, enhanced GABA_*A*_R-mediated miniature postsynaptic currents in hippocampal CA1 neurons (Zhang et al., 2016). Furthermore, exogenous E2 and the inhibitors of E2 synthesis or estrogen receptors exacerbated and alleviated, respectively, bumetanide-sensitive EEG-detectable seizures caused by neonatal anesthesia with sevoflurane (Zhang et al., 2016). Studies have demonstrated that GABAergic anesthetics, including sevoflurane, administered to neonatal rats can induce a persistent reduction of hippocampal and hypothalamic *Kcc2* expression (Ju et al., 2017, 2018). Such studies provide indirect support for the interaction between GABA_*A*_R signaling and E2 in the mediation of the neurodevelopmental effects of sevoflurane. Impairment in *Kcc2* expression may be mediated, at least in part, by sevoflurane-caused increases in E2 levels, as Galanopoulou and Moshé (2003) have shown that E2 lowered KCC2 levels in the developing male rat brain. The impaired *Kcc2* expression and resulting shift in GABA_*A*_R signaling to more excitatory may be a reason that the P9 to P11 rats that were neonatally exposed to sevoflurane showed increased susceptibility to sevoflurane-caused seizures.

Dysregulated neuroendocrine responses to stress, programmed by adverse life experiences, especially early in life, are an underlying feature of many neurodevelopmental and neuropsychiatric disorders (Lupien et al., 2009; Franklin et al., 2012; Berretz et al., 2020). In this study, we found that corticosterone responses to stress were exacerbated by neonatal exposure to sevoflurane, an effect that was sensitive to formestane. That finding suggests that sevoflurane, by acting through potentiation of E2 synthesis, can act as one such early life adverse experience capable of reprogramming neuroendocrine responses to stress. Such E2-dependent neurodevelopmental effects of neonatal exposure to sevoflurane are further supported by similar effects of formestane on sevoflurane-induced behavioral deficiencies revealed by the EPM and PPI of the startle tests. The EPM is a widely used assay to investigate anxiety/affective behavior in rodents (Walf and Frye, 2007; Schneider et al., 2011). The PPI of the startle is the reduction of the startle response when the startle stimulus is preceded by a subthreshold sensory stimulus (sensorimotor gating) (Koch and Schnitzler, 1997; Lauer et al., 2017; Fulcher et al., 2020). Impaired PPI of the startle has been demonstrated in patients with many neuropsychiatric and neurodevelopmental disorders, including schizophrenia and autism spectrum disorders (Takahashi and Kamio, 2018). PPI measurements in rodents, in contrast to almost all other rodent behavioral paradigms, can safely and easily be replicated under nearly identical parameters in humans (Rydkjaer et al., 2020). For that reason, the PPI of the startle is a promising experimental tool that may help to build a foundation for the much-needed mechanistic clinical studies in the field of the neurodevelopmental effects of general anesthetics. Similarly, measurement of heightened stress-related levels of cortisol, for example in saliva, may facilitate a transition in clinical studies from documentation of anesthesia-induced developmental neuroendocrine abnormalities to investigation of underlying mechanisms.

We have previously demonstrated that formestane at this dose, route, and timing of administration alleviated the acute adverse effects of sevoflurane in neonatal rats (Zhang et al., 2016). This was the main justification for testing its effects on the long-term neurodevelopmental effects of sevoflurane at the same dose, route, and timing of administration. Future mechanistic studies that include dose responses for aromatase and estrogen receptor modulators and measurements of levels of sex hormones will be needed to investigate the role of testosterone/estradiol in the neurodevelopmental effects of sevoflurane in male and female rats.

The results of this study demonstrate that the inhibitor of E2 synthesis formestane, similar to the inhibitor of the NKCC1 Cl^–^ importer bumetanide, diminished the ability of sevoflurane to induce persistent neurodevelopmental abnormalities in male rats. These results, along with our previously published findings (Cao et al., 2012; Xu et al., 2015; Zhang et al., 2016) suggest that the sevoflurane-caused increase in the synthesis of E2 and exacerbation of depolarizing/excitatory GABA_*A*_R signaling at the time of anesthesia may represent initial steps in the effects of the anesthetic to induce neurodevelopmental abnormalities in male rats. Future studies will be needed to determine whether similar mechanisms are functional in female rats. Future studies will also be needed to elucidate the interaction between the neurodevelopmental effects of general anesthetics and the adverse effects of accompanying surgeries and diseases. This work will help determine whether shorter exposures to general anesthetics in such settings can lead to more profound abnormalities.

## Data Availability Statement

The original contributions presented in the study are included in the article/supplementary material, further inquiries can be directed to the corresponding author/s.

## Ethics Statement

The animal study was reviewed and approved by the University of Florida Institutional Animal Care and Use Committee.

## Author Contributions

AM and JZ designed the research. JW, BY, LJ, JY, and AA performed the experiments and analyzed the data. JW, BY, and AM wrote the manuscript. All authors reviewed and edited the final version of the manuscript.

## Conflict of Interest

The authors declare that the research was conducted in the absence of any commercial or financial relationships that could be construed as a potential conflict of interest.
